# Active Autophagy Is Associated with Favorable Outcome in Patients with Surgically Resected Cholangiocarcinoma

**DOI:** 10.3390/cancers15174322

**Published:** 2023-08-29

**Authors:** Katrin Bankov, Falko Schulze, Steffen Gretser, Henning Reis, Nada Abedin, Fabian Finkelmeier, Jörg Trojan, Stefan Zeuzem, Andreas A. Schnitzbauer, Dirk Walter, Peter J. Wild, Maximilian N. Kinzler

**Affiliations:** 1 Dr. Senckenberg Institute of Pathology, University Hospital Frankfurt, Goethe University Frankfurt, 60590 Frankfurt am Main, Germany; 2 Department of Internal Medicine I, University Hospital Frankfurt, Goethe University Frankfurt, 60590 Frankfurt am Main, Germany; 3 Frankfurt Cancer Institute (FCI), University Hospital Frankfurt, Goethe University Frankfurt, 60590 Frankfurt am Main, Germany; 4 Department of General, Visceral, Transplant and Thoracic Surgery, University Hospital Frankfurt, Goethe University Frankfurt, 60590 Frankfurt am Main, Germany; 5 Frankfurt Institute for Advanced Studies (FIAS), 60438 Frankfurt am Main, Germany

**Keywords:** acetylation, autophagy, cholangiocarcinoma, surgical oncology, survival

## Abstract

**Simple Summary:**

Autophagy can exert a dual role in the context of cancer progression. However, data on the prevalence and impact of autophagy in primary cholangiocarcinoma (CCA) tissue are very limited. Active autophagy was present in a minority of the CCA patients (23.3%). We found a significantly impaired overall survival rate for patients with non-active autophagy (32.7 months) compared to CCA patients with active autophagy (68.4 months). In line with this, absence of active autophagy was an independent risk factor for overall survival. Moreover, in patients with active autophagy, pan-acetylation was significantly more prominent compared to those with non-active autophagy. Our data strengthen the role of active autophagy as a prognostically relevant marker and a potential therapeutic target in CCA patients.

**Abstract:**

Data on the impact of autophagy in primary cholangiocarcinoma (CCA) remain scarce. Here, we therefore investigated the role of active autophagy and its impact on survival in CCA patients. All CCA patients who underwent surgical resection with curative intent between 08/2005 and 12/2021 at University Hospital Frankfurt were evaluated. Autophagic key proteins were studied by immunohistochemistry. iCCA processed for gene expression profiling of immune-exhaustion gene sets was used for an autophagy approach in silico. Active autophagy was present in 23.3% of the 172 CCA patients. Kaplan–Meier curves revealed median OS of 68.4 months (95% CI = 46.9–89.9 months) and 32.7 months (95% CI = 23.6–41.8 months) for active and non-active autophagy, respectively (*p* ≤ 0.001). In multivariate analysis, absence of active autophagy (HR = 2, 95% CI = 1.1–3.5, *p* = 0.015) was an independent risk factor for OS. Differential-expression profiling revealed significantly upregulated histone deacetylases (HDAC) mRNA in patients showing non-active autophagy. In line with this, pan-acetylated lysine was significantly more prominent in CCA patients with ongoing autophagy (*p* = 0.005). Our findings strengthen the role of active autophagy as a prognostically relevant marker and a potential therapeutic target.

## 1. Introduction

Cholangiocarcinoma (CCA) represents a highly diverse form of cancer that originates from both the biliary epithelium within the liver (iCCA) and the bile ducts outside the liver parenchyma (eCCA). eCCA can be further categorized as perihilar (pCCA) or distal (dCCA) based on its localization. In terms of histologic characterization, there are two distinct subtypes of iCCA that have recently been introduced into the WHO classification, namely the small duct type (SD-iCCA) and the large duct type (LD-iCCA) [1,2]. The global incidence of this rare malignancy has steadily been increasing over the past few decades [3,4]. Despite surgical resection being the sole curative treatment option, the prognosis for CCA patients remains unfavorable [5]. For the vast majority of patients diagnosed at late stages of the disease, palliative chemotherapy with gemcitabine and cisplatin plus the PD-L1 inhibitor Durvalumab, leading to a median overall survival of 12.8 months, is the new standard of care [6,7]. Due to the poor outcomes for CCA patients, identification of alternative therapeutic approaches is of utmost importance to open new perspectives for this critically ill cohort.

Macroautophagy (hereinafter referred to as autophagy) is the major intracellular degradation system [8]. However, the aim of autophagy is not the simple elimination of excess or damaged cytoplasmic components, protein aggregates and organelles, but instead, autophagy is essential for maintaining cellular metabolism and homeostasis [8,9]. There is mounting evidence that autophagy can promote even contradictory effects in the context of cancer, resulting in the extensively discussed ‘dual role of autophagy’ [10,11,12]. Which is to say that cancer cells can harness the autophagic machinery to fulfil their enormous metabolic demands maintaining carcinogenesis or metastatic spread. In contrast, dysregulation of autophagy can contribute to tumor growth and progression, but can also impact antitumor immunity. As such, active autophagy is imperative for optimal anti-cancer immunosurveillance [13,14,15] linking autophagy’s potential to orchestrate oncogenic metabolism and inflammation.

Apart from few studies investigating the role of autophagic key players like protein 1 light chain 3 B (LC3B), sequestome 1 (p62) and pro-autophagic Beclin-1 in patients’ tissue by immunohistochemistry during cholangiocarcinogenesis [16] or in combined hepatocellular carcinoma (HCC)-CCA [17], studies determining the impact of autophagy in primary tumor tissue of CCA patients remain scarce so far. In more detail, three studies have shed light on the prognostic role of Beclin-1 in CCA [18,19,20] and two studies have assessed LC3B ± p62 only in iCCA [21,22], while no study has addressed the impact of active autophagy in all histopathological subtypes, including SD- and LD-iCCA.

Given the persistent poor outcomes for CCA patients even after surgical resection, there is significant clinical interest in exploring novel therapeutic strategies. In line with this, several studies highlight autophagy as a promising target in anti-cancer therapies. As such, it was shown that stimulation of autophagy impairs growth in human breast cancer, pancreatic cancer, and hepatocellular carcinoma cells [23,24,25]. In CCA patients, autophagy modulators, in combination with chemo-, immuno- or targeted therapies, could improve the therapeutic outcomes as well [26].

In summary, this study provides a comprehensive assessment of the impact of active autophagy on survival of surgically resected CCA patients, encompassing all histopathologic subtypes.

## 2. Materials and Methods

### 2.1. Study Population

The study builds upon the patient population and data base of our previously established tissue microarray (TMA) cohort of CCA patients [27], while histological confirmation of SD-iCCA and LD-iCCA cases was confirmed as well [28]. The University Cancer Center Frankfurt (UCT) provided the tissue samples utilized in this study. Prior to their inclusion, written informed consent was obtained from all patients, and the study received approval from the institutional Review Boards of both the UCT and the Ethical Committee at the University Hospital Frankfurt (project-number: SGI-13-2018).

### 2.2. Immunohistochemistry (IHC)

TMA construction was performed as previously reported [27]. Staining of LC3B (polyclonal; dilution: 1:500; incubation time: 30 min; NovusBio, Centennial, CO, USA), p62 (Clone: EP396; dilution: 1:200; incubation time: 30 min; Abcam, Cambridge, UK), Beclin-1 (monoclonal; dilution: 1:100; incubation time: 30 min; REF#AC-0276, Abcam, Cambridge, UK) and pan-acetylated-lysine (polyclonal; dilution: 1:1000; incubation time: 30 min; REF#9441, Cell Signaling Technology, Danvers, MA, USA) was conducted. Stained slides were scanned with the Pannoramic slide scanner (3DHISTECH, Budapest, Hungary). Evaluation of the IHC staining was performed semi-quantitatively by determining the percentage of positively stained tumor cells (0: <5%, 1: 5–25%, 2: 26–75%, 3: >75%) and the staining intensity (0: none/background, 1: weak, 2: moderate, 3: strong). Due to stronger background staining for LC3B, IHC scores ≤4 and ≥5 were considered low and high, respectively. For p62, Beclin-1 and pan-acetylated-lysine, IHC scores ≤3 and ≥4 were considered low and high, respectively. IHC analysis was performed by two independent investigators. In line with literature, expression of LC3B high/p62 low was defined as ‘active autophagy’ [22,29]. TMA cores lacking representative tumor tissue or exhibiting staining artifacts were excluded from the analysis.

### 2.3. Ribonucleic Acid (RNA) Isolation and Immune-Exhaustion Expression Analysis

RNA isolation was conducted as described recently [27]. Immune-exhaustion expression analysis was performed using the Nanostring nCounter^®^ Platform, the Immune Exhaustion Panel v1, Nanostring nSolver™ software v4 as well as the nCounter^®^ Advanced Analysis module v2.0.115 (Nanostring, Seattle, WA, USA), as indicated previously [27]. iCCA patients were classified into two groups based on the presence or absence of active autophagy, as determined by prior IHC analysis. To identify enriched genes associated with overrepresented gene ontologies based on *REACTOME_pathways* (updated 15 November 2022), functional classification and network annotation were performed using ClueGO v.2.5.6 and CluePedia v.1.5.6. [30,31].

### 2.4. Statistical Analysis

Clinicopathological characteristics were compared between patients with and without active autophagy, while categorical and continuous variables were analyzed as indicated recently [27]. Definition and statistical analysis of overall survival (OS), including Kaplan–Meier curves and Cox regression analysis, was performed as previously described [27]. A significance level of *p* < 0.05 was used for determining statistical significance. The data analysis was conducted using SPSS 27 (IBM; Armonk, BY, USA) statistical software and GraphPad Prism v.9.5.1. Violin plots, scatter plots, chord plots and heatmap were computed by https://www.bioinformatics.com.cn/en, accessed on 20 August 2023, a free online platform for data analysis and visualization.

## 3. Results

### 3.1. Patients and Clinical Characteristics

**Figure 1:**
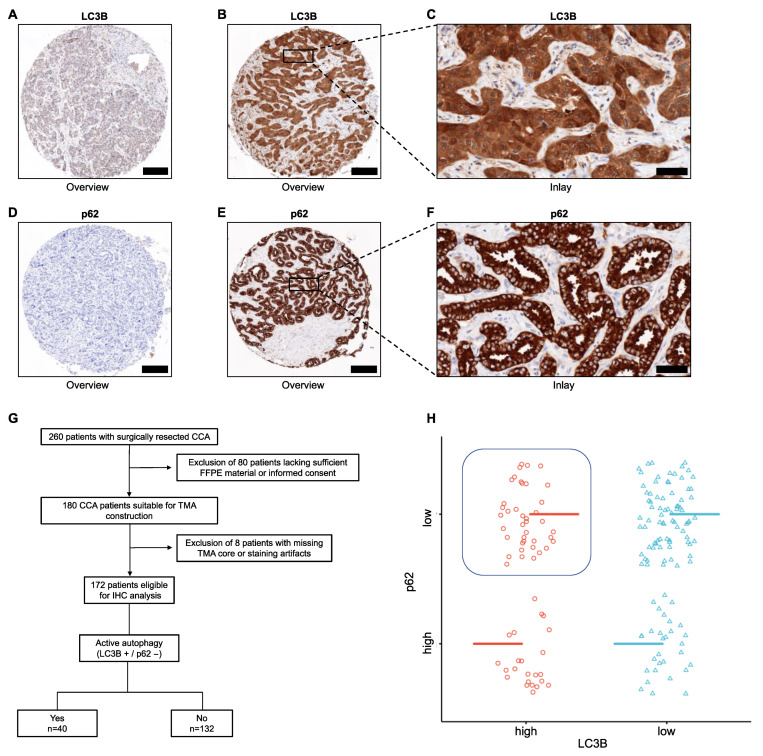
Representative images of LC3B and p62 expression in CCA and inclusion workflow. (**A**–**C**) Representative immunohistochemistry of low (**A**) and high (**B**,**C**) expression of LC3B in TMA cores of CCA patients. (**D**–**F**) Representative immunohistochemistry of low (**D**) and high (**E**,**F**) expression of p62. Original magnification ×8.5 for overview and ×40 for inlay, respectively. Scale bars: 200 µm for overview and 50 µm for inlay, respectively. (**G**) Inclusion workflow for patients showing active and non-active autophagy. (**H**) Scatter plot showing the distribution of low and high expression of LC3B and p62 in CCA tissue, respectively. The overlap of LC3B high and p62 low is highlighted, as it indicates the proportion of cases with active autophagy. (Line-marking of the categorical value and applied jittering improve data point visibility). Abbreviations: cholangiocarcinoma (CCA), immunohistochemistry (IHC), tissue microarray (TMA).

In total, 172 patients in our tertiary hospital with surgically resected CCA were suitable for IHC analysis after TMA construction. Representative IHC images of both low and high expression of LC3B and p62 are shown in Figure 1A–F. Of the enrolled patients, 23.3% (*n* = 40) showed autophagic activity, while 76.7% (*n* = 132) did not (Figure 1G,H). CCA patients with active autophagy had lower pathological grading (*p* = 0.009) and better performance status (*p* = 0.026). In contrast, patients without autophagic activity had larger tumor size (*p* = 0.004) and a more frequent occurrence of multiple tumors (*p* = 0.025). Further clinicopathological characteristics are depicted in Table 1. In addition to the assessment of active autophagy, high expression of Beclin-1 was present in 46.2% of CCA patients. Representative IHC images of low and high expression of Beclin-1 are shown in Figure S1A,B.

### 3.2. Impact of Autophagic Activity on Overall Survival

**Figure 2:**
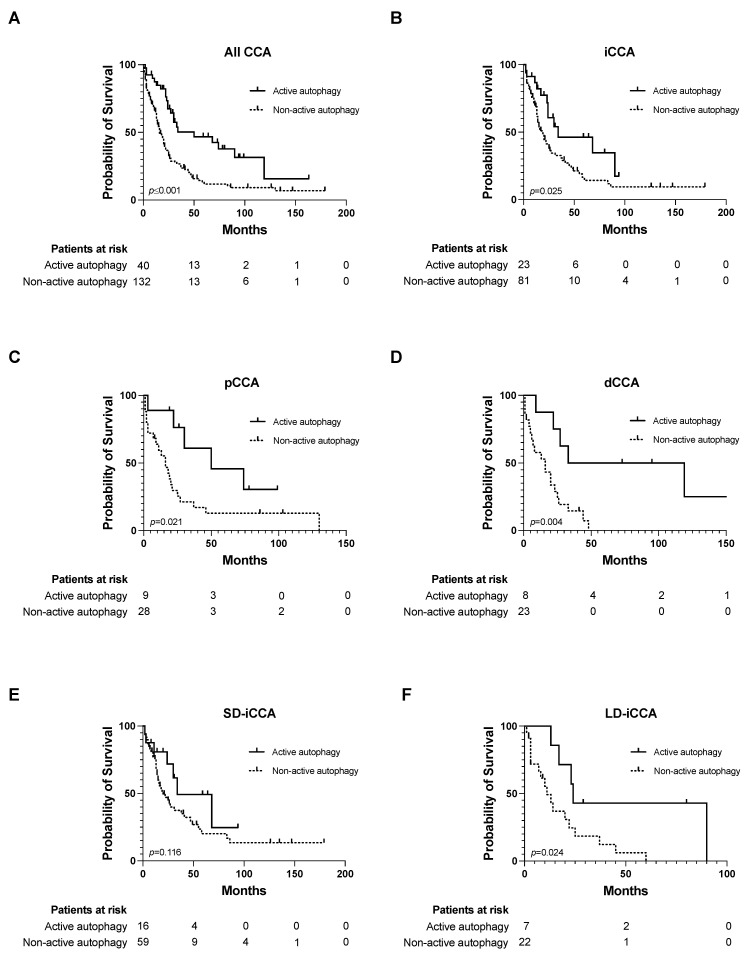
Kaplan–Meier curves for overall survival in CCA patients showing active and non-active autophagy. (**A**–**F**) Overall survival investigated for active autophagy in all types of CCA (**A**), iCCA (**B**), pCCA (**C**), dCCA (**D**), small duct type iCCA (**E**) and large duct type iCCA (**F**). Date of last follow-up was treated as censored observation. Abbreviations: cholangiocarcinoma (CCA), intrahepatic cholangiocarcinoma (iCCA), distal cholangiocarcinoma (dCCA), large duct type iCCA (LD-iCCA), perihilar cholangiocarcinoma (pCCA), small duct type iCCA (SD-iCCA).

As the presence of autophagic activity was linked to favorable tumor features, our objective was to examine its impact on OS within our cohort. Kaplan–Meier curves revealed a median OS of 68.4 months (95% CI = 46.9–89.9 months) for all CCA patients displaying active autophagy, in comparison to 32.7 months (95% CI = 23.6–41.8 months) for patients lacking effective autophagy (*p* ≤ 0.001) (Figure 2A). In iCCA, OS rates were 50.5 months (95% CI = 34.2–66.9) and 38.4 months (95% CI = 25.3–52.5) for presence and absence of autophagic activity, respectively (*p* = 0.025) (Figure 2B). For pCCA patients with present and absent autophagy, the median OS was 56.8 months (95% CI = 32.1–81.4) and 26.1 months (95% CI = 11–41.2), respectively (*p* = 0.021) (Figure 2C). Correspondingly, active autophagy is associated with prolonged OS rates in patients with dCCA (81.9 months (95% CI = 37.9–125.8)) in contrast to patients without autophagic activity (16.8 (95% CI = 10.4–23.2)) (*p* = 0.004) (Figure 2D). We also investigated, for the first time, the impact of active autophagy in SD-iCCA and LD-iCCA. In SD-iCCA, OS rates were 50.3 months (95% CI = 30.1–70.5) and 46.6 months (95% CI = 29.2–64) for presence and absence of autophagic activity, respectively (*p* = 0.116) (Figure 2E). In contrast, OS rates were significantly prolonged in LD-iCCA patients showing autophagic activity (49.6 months (95% CI = 20.4–78.7) compared to 17.3 months (95% CI = 10–25.1) (*p* = 0.024)) (Figure 2F). Next, we investigated the impact of high Beclin-1 expression in CCA tissue on OS in our study cohort. High expression of Beclin-1 was also associated with prolonged OS for all CCA, although this observation did not reach statistical significance (*p* = 0.088). Importantly, prolonged OS rates in patients with high Beclin-1 expression were demonstrated for iCCA (*p* = 0.037) and pCCA (*p* = 0.044), while no improved OS rates could be shown for dCCA patients (*p* = 0.303) (Figure S1C–F).

### 3.3. Active Autophagy as an Independent Risk Factor for OS

Next, we conducted both univariate and multivariate Cox regression analysis to determine potential risk factors associated with a poor outcome. Importantly, the univariate analysis revealed that the absence of autophagic activity is a significant risk factor of OS (HR = 2.5, 95% CI = 1.5–3.89, *p* ≤ 0.001). Moreover, positive CA-19/9 (HR = 2.2, 95% CI = 1.5–3.3, *p* ≤ 0.001), multiple tumors (HR = 2.1, 95% CI = 1.4–3, *p* ≤ 0.001), pathological grade 3 (HR = 4.6, 95% CI = 1.1–19.2, *p* = 0.036), lower performance status (HR = 2.5, 95% CI = 1.7–3.8, *p* ≤ 0.001), M1 status (HR = 2.6, 95% CI = 1.4–4.7, *p* = 0.002) and elevated serum bilirubin (HR = 1.6, 95% CI = 1.1–2.2, *p* = 0.019) also served as significant risk factors in univariate analysis. Multivariate analysis indicated that absence of active autophagy (HR = 2, 95% CI = 1.1–3.5, *p* = 0.015), ECOG 1 (HR = 2.2, 95% CI = 1.4–3.5, *p* = 0.001), CA-19/9 (HR = 1.8, 95% CI = 1.2–2.8, *p* = 0.007), M1 status (HR = 2.1, 95% CI = 1–4.2, *p* = 0.049), as well as presence of elevated serum bilirubin (HR = 1.6, 95% CI = 1–2.4, *p* = 0.04) serve as independent risk factors for OS for CCA patients in this cohort (Table 2). Of note, Beclin-1 did not serve as a significant risk factor for OS in all CCA in univariate testing (*p* = 0.094).

### 3.4. iCCA with Active Autophagy Displays a Differential Expression Profile

**Figure 3:**
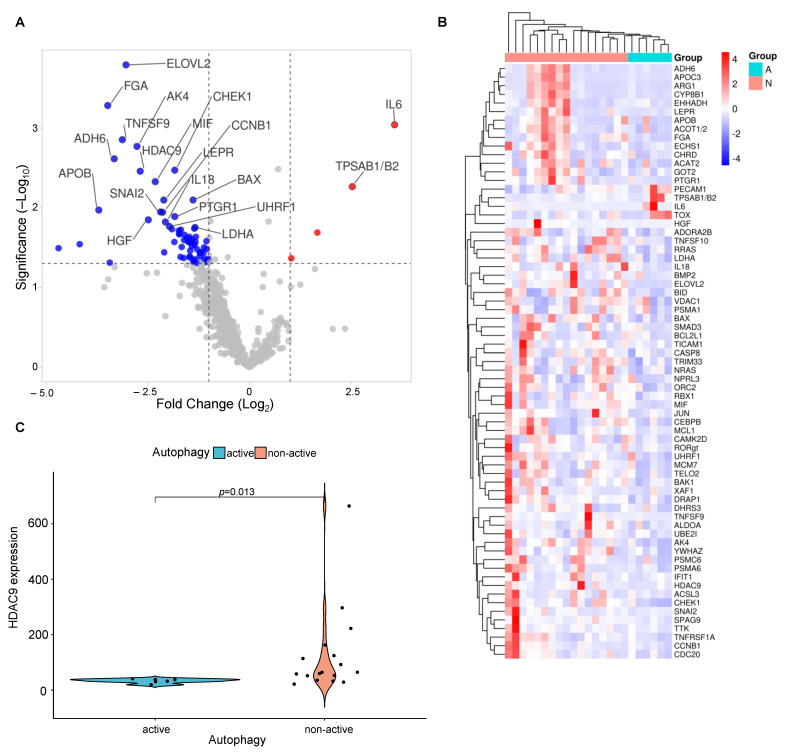
Prominently deregulated transcripts of active vs. non-active autophagy in iCCA. (**A**) Volcano plot showing each gene’s -log10 (*p*-Value) and log2 fold change with the selected covariate [32]. Highly statistically significant genes are positioned above the horizontal lines, while highly differentially expressed genes are distributed on either side. The plot includes labels for the 20 most statistically significant genes. (**B**) Unsupervised hierarchical clustering of differentially expressed genes among iCCA groups of active vs. non-active states of autophagy using Manhattan distance method and average linkage clustering across samples and transcripts. Active autophagy (*n* = 6), non-active autophagy (*n* = 17). Blue color indicates low expression, red color indicates high expression. (**C**) HDAC9-mRNA expression among groups of active and non-active autophagy. Violin plot with additional scatter plot (jittered values) presenting HDAC9-mRNA expression among groups tested for significance by the Wilcoxon rank-sum test (*p* = 0.013). Abbreviations: active autophagy (Group A), non-active autophagy (Group N).

Our previously established cohort of 23 cases of iCCA processed for gene expression profiling of immune-exhaustion gene sets was utilized for our autophagy approach in silico [27]. By means of the applied IHC scoring system, the cases were stratified into groups of active (6/23) and non-active autophagy (17/23) and analyzed to scrutinize expression changes. Our data revealed 69 genes as differentially regulated between the two stratified groups with active and non-active autophagy (log2 fold change (FC) larger than 1 (linear FC ≥ 2); *p*-Value ≤ 0.05). Due to a high false discovery rate (FDR) and limited sample size, the application of a *p*-value correction according to the Bonferroni–Yekuteli method was not feasible. The most differentially expressed genes are displayed in a volcano plot and highlighted according to their significance (e.g., upregulated IL6; downregulated histone deacetylase (HDAC) and BAX) (Figure 3A–C, Table S1).

**Figure 4:**
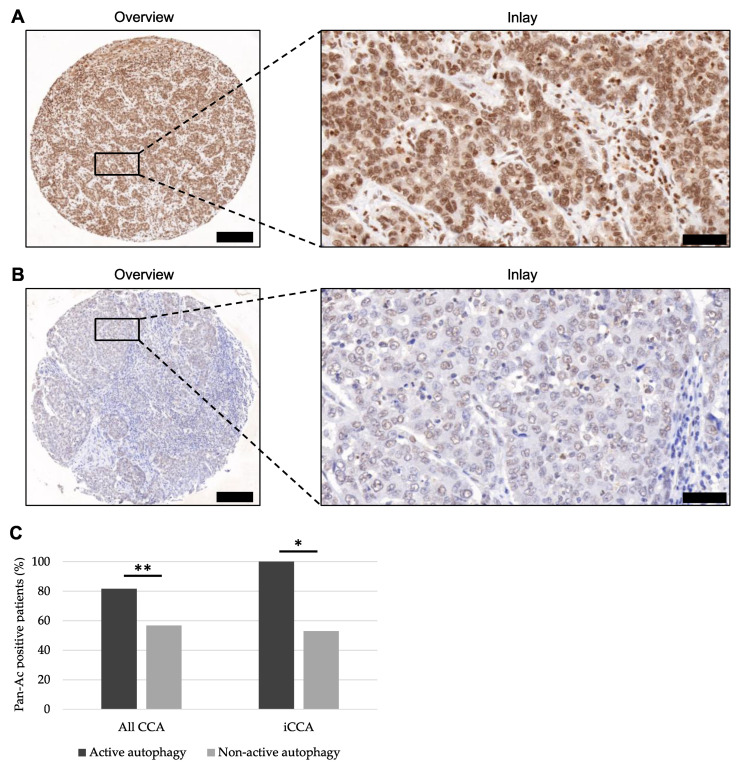
Representative images of pan-acetylated-lysine antibody staining in CCA tissue and expression differences between the groups of active and non-active autophagy. (**A**,**B**) Representative immunohistochemistry of high (**A**) and low (**B**) expression of pan-acetylated-lysine in TMA cores of CCA patients. Original magnification ×8.5 for overview and ×40 for inlay, respectively. Scale bars: 200 µm for overview and 50 µm for inlay, respectively. (**C**) Differences in pan-Ac expression between CCA patients showing active and non-active autophagy for all CCA subtypes and iCCA, respectively. Significance determinations were calculated using Spearman correlation as a measure of a variable’s association. *p*-Values were interpreted as follows: * *p* < 0.05; ** *p* ≤ 0.01. Abbreviations: cholangiocarcinoma (CCA), intrahepatic cholangiocarcinoma (iCCA), pan-acetylated-lysine (pan-Ac), tissue microarray (TMA).

Next, we performed verification of downregulated histone deacetylases as potential clinically relevant targets by assessing the pan-acetylation status as a surrogate marker via IHC analysis. Representative images of high and low expression of pan-acetylated-lysine in CCA tissue are depicted in Figure 4A,B. Consistent with our expression data, IHC scoring showed 100% and 53% pan-acetylated-lysine-positive iCCA tissue in patients with active- and non-active autophagy, respectively. Thus, the pan-acetylation status differed significantly between both groups (*p* = 0.039). To further confirm these preliminary data, we evaluated the pan-acetylation status for all CCA in our TMA cohort. Our results suggest 81.6% and 56.7% pan-acetylated lysine-positive CCA tissue in patients with active- and non-active autophagy, respectively. Hence, pan-acetylation was significantly more prominent in CCA patients with ongoing autophagy (*p* = 0.005) (Figure 4C).

**Figure 5:**
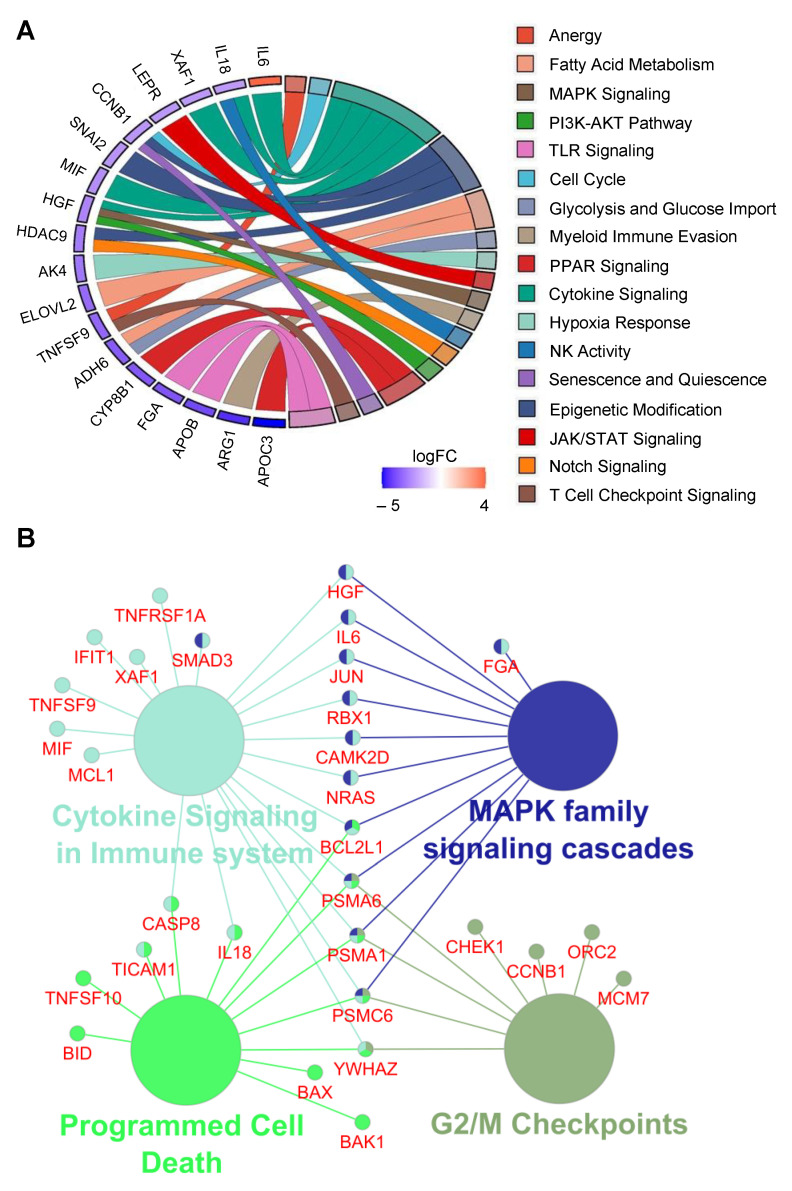
Gene sets reveal functional classification. (**A**) Chord plot summarizing the most prominent gene sets, which are displayed with their associated genes being significantly deregulated (*p*-Value ≤ 0.05, −2 ≥ log2FC ≥ 2); additionally, the log2FC is encoded by color at each candidate gene, as indicated by the legend (red color, upregulated; blue color, downregulated). (**B**) Top 30 statistically overrepresented genes assigned to terms such as ‘Cytokine Signaling in Immune System’, ‘MAPK family signaling cascades’, ‘Programmed Cell Death’ and ‘G2/M Checkpoints’ using the *REACTOME_pathways* ontology database.

Top associated gene sets revealed theory-matching terms like Fatty Acid Metabolism, Apoptosis, and Antigen Presentation, as well as Hypoxia Response and PI3K-AKT Pathway. The identified terms and differentially regulated transcripts are shown in a chord plot (Figure 5A).

Functional classification and pathway annotation of the aforementioned 69 transcripts highlighted overrepresented altered gene ontologies such as G2/M checkpoint (e.g., CCNB1 and CHEK1, among others), programmed cell death (e.g., BAK1 and BAX, among others), cytokine signaling in immune system highlighting interleukins, among others already known for their association in immunogenic cell death (e.g., IL6, IL8), and MAPK-family signaling cascades (e.g., NRAS, JUN, among others). The pathway network and a selection of their interacting genes provide a first tentative insight into the molecular mechanisms in CCA showing active autophagy (Figure 5B, Table S2).

## 4. Discussion

The clinical relevance of autophagy in carcinogenesis, cancer progression and as a potential target for novel therapeutic approaches in CCA is just beginning to emerge. Assessment of autophagy in the primary tumor tissue of CCA patients is mainly based on immunostaining of single autophagic proteins, while the role of active autophagy has only been studied to a very limited extent. This study is the first to determine the incidence and impact of active autophagy on survival in all histological subtypes of CCA by immunostaining and differential expression profiling in order to provide tentative insights beyond the mere expression.

In the present study, active autophagy could be observed in 23.3% of the patients, whereas it was absent in the majority of CCA cases. In addition to the prevalence of ongoing autophagy in our cohort, this study also sheds light on its impact on the clinical course. Our study showed a substantial increase in overall survival rates in patients with active autophagy (68.4 months) compared to CCA patients without effective autophagy (32.7 months). There are several aspects that warrant discussion as potential co-factors contributing to the beneficial effects of ongoing autophagy on survival in our study. In short, patients with ongoing autophagy had lower pathological grading, better performance status, smaller tumor size and less frequently had multiple tumors. Importantly, the absence of active autophagy remains an independent risk factor associated with shortened survival as well as a lower performance status, positive CA-19/9, distant metastasis and elevated levels of serum bilirubin.

Assessment of autophagy in primary tumor tissue is generally performed by determining indirect markers like LC3B, p62 or Beclin-1. The cytosolic LC3B, which is among the best-studied proteins for monitoring canonical autophagy, adopts a membrane-bound state after lipidation and becomes anchored to the growing autophagosome [33]. LC3B then actively promotes the engulfment of bulk cytoplasmic content or selective cellular components through its binding to adaptor proteins like p62, which are degraded in the resulting autophagolysosomes [34]. Hence, the combined pattern of LC3B high/p62 low indicates proceeding and effective autophagy [8,20,29]. In contrast, LC3B low/p62 low represents basal autophagy, whereas LC3B low/p62 high and LC3B high/p62 high represent impaired autophagy at early and late stages, respectively [22,29]. Next, the pro-autophagic Beclin-1 complex is generally involved in the initiation of autophagosome formation, serving as a widely used autophagy marker. As the dynamic process of autophagy is best reflected by the combined evaluation of LC3B high and p62 low [8], we mainly elucidated the impact of active autophagy in our cohort. In addition, the role of Beclin-1 was investigated as a third autophagy-related key protein.

In line with our data, Thongchot et al. recently determined that ongoing autophagy was associated with longer survival in a TMA cohort containing 70 iCCA [22]. Remarkably, we describe a similar proportion of patients with effective autophagy compared to Thongchot et al. (28.6%), although these data solely refer to iCCA [22]. However, it needs to be considered that the latter study was based on an Asian cohort in which six cases received neoadjuvant chemotherapy before surgical resection, and stratification into SD-iCCA and LD-iCCA was lacking [22]. Importantly, the higher prevalence of biliary parasites, as an important risk factor for CCA development in Asia, might affect the comparability between the two cohorts. Hence, our study is the first investigating the impact of active autophagy in a European cohort. In contrast to the evaluation of combined LC3B high/p62 low indicative for ongoing autophagy, several studies investigated the prognostic role of single autophagic key players in CCA tissue. In the present study, we observed a slightly higher proportion of Beclin-1 positive CCA patients (46.2%) compared to data from Wang et al. (30%) [19]. Our data indicate that high Beclin-1 expression is associated with significantly prolonged OS rates in iCCA and pCCA. These observations are consistent with the findings of Wang et al., who described improved OS rates for iCCA patients with high Beclin-1 expression, but not for all CCA in general [19]. In line with these data, Dong et al. reported a longer overall and disease-free survival rate for iCCA patients highly expressing Beclin-1 in primary tumor tissue [18]. Next, Lendvai et al. suggested a beneficial outcome for dCCA patients expressing enhanced levels of Beclin-1 [20]. Intriguingly, Beclin-1 expression correlated positively and negatively with LC3B and p62, respectively [20]. Thus, we hypothesize that the prognostic role of Beclin-1 in dCCA in the study by Lendvai et al. is based on active autophagy as well. Importantly, we describe prolonged OS rates for active autophagy in dCCA, but not for high Beclin-1 expression. However, the very small sample size and bias in the selection of dCCA patients in the study by Lendvai et al. (*n* = 23) and our study cohort (*n* = 30) may hamper comparability [20]. Moreover, bypassing canonical autophagy through independency of Beclin-1 is the most prominent non-canonical pathway shedding light on the heterogenous initiation process of autophagosome formation and the difficulty of studying the autophagic process by assessing single proteins [35,36].

Furthermore, Chen et al. recently suggested high expression of LC3B as an unfavorable predictor for OS in an Asian iCCA cohort [21]. However, determination of Beclin-1 or LC3B alone may indicates that autophagy has been initiated, but it remains elusive whether autophagy proceeds effectively or is impaired [9]. Here, we provide conclusive data on active autophagy in all CCA subtypes from one cohort of our tertiary hospital, strengthening the view of the beneficial role of autophagy as a prognostically relevant marker. We encourage future studies to investigate the prognostic role of autophagy in CCA by assessing combined autophagic key proteins that better reflect the dynamic process of autophagy.

During recent years it has been indicated that autophagy can either serve tumor-suppressive or oncogenic functions in the context of cancer [37]. On the one hand, autophagy can protect malignant conditions from nutrient deprivation, or even by hindering anti-cancer therapy. As such, it is widely reported that active autophagy fuels resistance to therapeutical agents such as bortezomib and sorafenib, as well as immune checkpoint inhibitors [38,39,40]. Intriguingly, it has been suggested that inhibition of active autophagy increases sensitivity to cisplatin in CCA as well [41]. On the other hand, it is of mere interest that we have stratified a majority of tissues lacking active autophagy while ongoing autophagy is linked with beneficial patient outcome in our study. However, one should consider that our study is based on a CCA cohort undergoing surgical resection with curative intent whereas the impact of autophagy on treatment outcome, e.g., in recurrent patients, was not investigated separately. Nonetheless, we speculate that patients lacking active autophagy may better respond to systemic therapy as well. Since the majority of CCA patients are irresectable when diagnosed, studies addressing the role of active autophagy and its impact on different chemo-/immunotherapies in the palliative setting are urgently needed. As our data indicate a beneficial role of autophagy in CCA patients, several therapeutic approaches need to be discussed. First, BCL-2 homology 3 (BH3) mimetics were shown to induce autophagy by disrupting the inhibitory interference between Beclin-1 and BCL-2 family proteins, thus liberating pro-autophagic Beclin-1 [42]. In line with this, our data reflected a higher expression of apoptosis-related transcripts in the cohort stratified to non-active autophagy (BAK1, BAX, BID, BCL2L1, MCL1, CASP8, TNFSF10). However, only one study has investigated the role of BH3 mimetics in human CCA cell lines so far [43]. A second mechanism reflected by our data is the higher portion of HDAC9 mRNA within the group of patients showing non-active autophagy. Several studies proved that HDAC inhibition results in inactivation of the mechanistic target of rapamycin (mTOR), thus promoting autophagy [44,45], while acetylation is described as a mechanistic switch in favor of anti-cancer processes in HCC and CCA in vitro [46,47,48]. In line, our expression data suggested high levels of HDAC9 in patients lacking active autophagy, while this group displays impaired OS rates. Next, these preliminary results were validated on the protein level as we showed significantly less pan-acetylation in this cohort. Therefore, we hypothesize that epigenetic modifications, such as acetylation, played a pivotal role in our CCA cohort and influenced patient survival. These data may have marked clinical implications, as stimulation of autophagy via HDAC inhibition might exert anticancer effects in CCA patients, including chemosensitivity [45,47]. This study is the first to provide a preliminary link between HDAC, acetylation status, and autophagy in primary tumor tissues from CCA patients, which may pave the way for new therapeutic approaches.

The present study has several limitations that should be acknowledged. First, the retrospective, single-center study design represents a major limitation. This design may introduce case selection bias, particularly for pCCA, dCCA and LD-iCCA. However, it is important to note that despite the relatively modest sample size, the number of CCA cases in our cohort is notably larger compared to similar studies using CCA-TMAs [20,22]. Second, it should be considered that no statement can be made as to tumor heterogeneity, as TMA cores represent only a small fraction of the total tumor tissue. Third, one should acknowledge that this study serves as an initial exploration into the genes that exhibit remarkable deregulation between the presence and absence of active autophagy. Importantly, this analysis was performed on a limited case series and focuses exclusively on iCCA. Therefore, no comprehensive confirmatory testing was held except for HDAC9 deregulation, which was addressed by a surrogate marker via IHC. Moreover, mechanistic testing by in vitro models was not applied, but needs to be addressed in future studies. Fourth, as a common scoring system for the analysis of the autophagic key proteins by immunohistochemistry is still lacking, determining the applicability of these antibodies could be challenging indeed, especially in clinical routine practice.

## 5. Conclusions

This study is the first analyzing the expression of activated autophagy in all histological CCA subtypes and its impact on survival. Active autophagy is present in the minority of CCA patients. In the group of patients with active autophagy, an improvement in survival was observed. These findings strengthen the presence of active autophagy as a prognostically relevant marker and should encourage future studies focusing on modulators of autophagy as therapeutic targets in patients suffering from CCA.

## Figures and Tables

**Table 1:** Baseline characteristics.

	Active Autophagy		
Characteristics	Yes (*n* = 40) No. (%)	No (*n* = 132) No. (%)	*p*-Value
Sex			0.573
Female	12 (30)	46 (34.8)	
Male	28 (70)	86 (65.2)	
Age at initial diagnosis			0.318
Mean, years, (range)	64.1 (42–86)	65.9 (38–86)	
CCA subtype			0.649
iCCA	23 (57.5)	81 (61.4)	
pCCA	9 (22.5)	28 (21.2)	
dCCA	8 (20)	23 (17.4)	
iCCA subtype			0.76
Small duct	16 (69.6)	59 (72.8)	
Large duct	7 (30.4)	22 (27.2)	
ECOG			0.026
0	32 (80)	82 (62.1)	
1	8 (20)	45 (34.1)	
2	0 (0)	5 (3.8)	
CA-19/9 (ng/mL)			0.23
<37	16 (40)	46 (34.8)	
≥37	13 (32.5)	62 (47)	
n.a.	11 (27.5)	24 (18.2)	
Tumor size (cm)			0.004
≤5	31 (77.5)	69 (52.3)	
>5	9 (22.5)	63 (47.7)	
Single Tumor			0.025
Yes	33 (82.5)	84 (63.6)	
No	7 (17.5)	48 (36.4)	
Pathological grade			0.009
Grade 1	2 (5)	1 (0.8)	
Grade 2	33 (82.5)	91 (68.9)	
Grade 3	5 (12.5)	40 (30.3)	
M status			0.883
M0	37 (92.5)	123 (93.2)	
M1	3 (7.5)	9 (6.8)	
R status			0.063
R0	35 (87.5)	93 (70.5)	
R1	5 (12.5)	34 (25.8)	
Rx	0 (0)	5 (3.8)	
L status			0.297
L0	22 (55)	58 (43.9)	
L1	13 (32.5)	52 (39.4)	
Lx	5 (12.5)	22 (16.7)	
Pn status			0.119
Pn0	15 (37.5)	32 (24.2)	
Pn1	19 (47.5)	76 (57.6)	
Pnx	6 (15)	24 (18.2)	
Recurrence			0.723
Yes	16 (40)	57 (43.2)	
No	24 (60)	75 (56.8)	
Cholelithiasis			0.746
Yes	3 (7.5)	8 (6.1)	
No	37 (92.5)	124 (93.9)	
PSC			0.172
Yes	0 (0)	6 (4.5)	
No	40 (100)	126 (95.5)	
Viral hepatitis			0.071
Yes	6 (15)	8 (6.1)	
No	34 (85)	124 (93.9)	
Diabetes			0.286
Yes	7 (17.5)	34 (25.8)	
No	33 (82.5)	98 (74.2)	
Liver cirrhosis			0.905
Yes	2 (5)	6 (4.5)	
No	38 (95)	126 (95.5)	
LDH			0.732
<248	20 (50)	68 (51.5)	
≥248	8 (20)	32 (24.2)	
n.a.	12 (30)	32 (24.2)	
Bilirubin			0.551
<1.4	28 (70)	86 (65.2)	
≥1.4	11 (27.5)	43 (32.6)	
n.a.	1 (2.5)	3 (2.3)	

The incidence levels of SD- and LD-iCCA were calculated relative to the total number of iCCA. Abbreviations: carbohydrate antigen 19-9 (CA-19/9), Eastern Cooperative Oncology Group (ECOG), intrahepatic cholangiocarcinoma (iCCA), distal cholangiocarcinoma (dCCA), lactate dehydrogenase (LDH), not available (n.a.), number (No.), perihilar cholangiocarcinoma (pCCA), primary sclerosing cholangitis (PSC).

**Table 2:** Cox regression analysis.

	Univariate Analysis			Multivariate Analysis		
Characteristics	HR	95% CI	*p*-Value	HR	95% CI	*p*-Value
Sex						
Female	ref					
Male	1.232	0.846–1.794	0.276			
CCA subtype						
iCCA	ref					
pCCA	1.228	0.8–1.885	0.348			
dCCA	1.234	0.786–1.937	0.36			
ECOG						
0	ref			ref		
1	2.547	1.724–3.763	<0.001	2.209	1.403–3.476	0.001
2	2.386	0.869–6.554	0.092	1.684	0.588–4.829	0.332
CA-19/9 (ng/mL)						
<37	ref			ref		
≥37	2.197	1.455–3.317	<0.001	1.814	1.176–2.799	0.007
Tumor size (cm)						
≤5	ref					
>5	1.058	0.742–1.508	0.755			
Single Tumor						
Yes	ref			ref		
No	2.09	1.438–3.039	<0.001	1.522	0.982–2.36	0.06
Active autophagy						
Yes	ref			ref		
No	2.46	1.546–3.913	<0.001	1.988	1.143–3.459	0.015
Pathological grade						
Grade 1	ref			ref		
Grade 2	1.616	0.397–6.584	0.503	0.842	0.111–6.381	0.868
Grade 3	4.596	1.101–19.182	0.036	1.902	0.246–14.7	0.538
M status						
M0	ref			ref		
M1	2.555	1.393–4.686	0.002	2.05	1.004–4.184	0.049
R status						
R0	ref					
R1	1.435	0.963–2.137	0.076			
Recurrence						
No	ref					
Yes	1.051	0.74–1.494	0.781			
Cholelithiasis						
No	ref					
Yes	1.643	0.881–3.067	0.119			
PSC						
No	ref					
Yes	1.651	0.725–3.759	0.232			
Diabetes						
No	ref					
Yes	1.197	0.794–1.804	0.39			
Viral hepatitis						
No	ref					
Yes	0.512	0.239–1.099	0.086			
Liver cirrhosis						
No	ref					
Yes	0.713	0.263–1.935	0.507			
LDH						
<248	ref					
≥248	1.211	0.779–1.884	0.396			
Bilirubin						
<1.4	ref			ref		
≥1.4	1.55	1.075–2.235	0.019	1.555	1.02–2.369	0.04

Abbreviations: carbohydrate antigen 19-9 (CA-19/9), cholangiocarcinoma (CCA), confidence interval (CI), Eastern Cooperative Oncology Group (ECOG), hazard ratio (HR), intrahepatic cholangiocarcinoma (iCCA), distal cholangiocarcinoma (dCCA), lactate dehydrogenase (LDH), perihilar cholangiocarcinoma (pCCA), primary sclerosing cholangitis (PSC).

## Data Availability

The data that support the findings of this study are available from the corresponding author upon reasonable request.

## Supplementary Materials

The following supporting information can be downloaded at: https://www.mdpi.com/article/10.3390/cancers15174322/s1. Figure S1: Representative images of Beclin-1 antibody staining in CCA tissue and corresponding Kaplan–Meier curves for overall survival; Table S1: The most differentially-expressed genes among iCCA groups of active vs. non-active states of autophagy; Table S2: Top genes from all clusters associated with nine representative terms and pathways using the *REACTOME_pathways* ontology database.
