# Supplementary material for: Active Autophagy Is Associated with Favorable Outcome in Patients with Surgically Resected Cholangiocarcinoma
Source: Cancers (Basel). 2023 Aug 29;15(17):4322. doi: 10.3390/cancers15174322 (PMC10486413; doi:10.3390/cancers15174322)
Supplement: Supplementary file 1 [file cancers-15-04322-s001.zip › cancers-2468463-supplementary.pdf]

Supplementary Figures

Figure S1

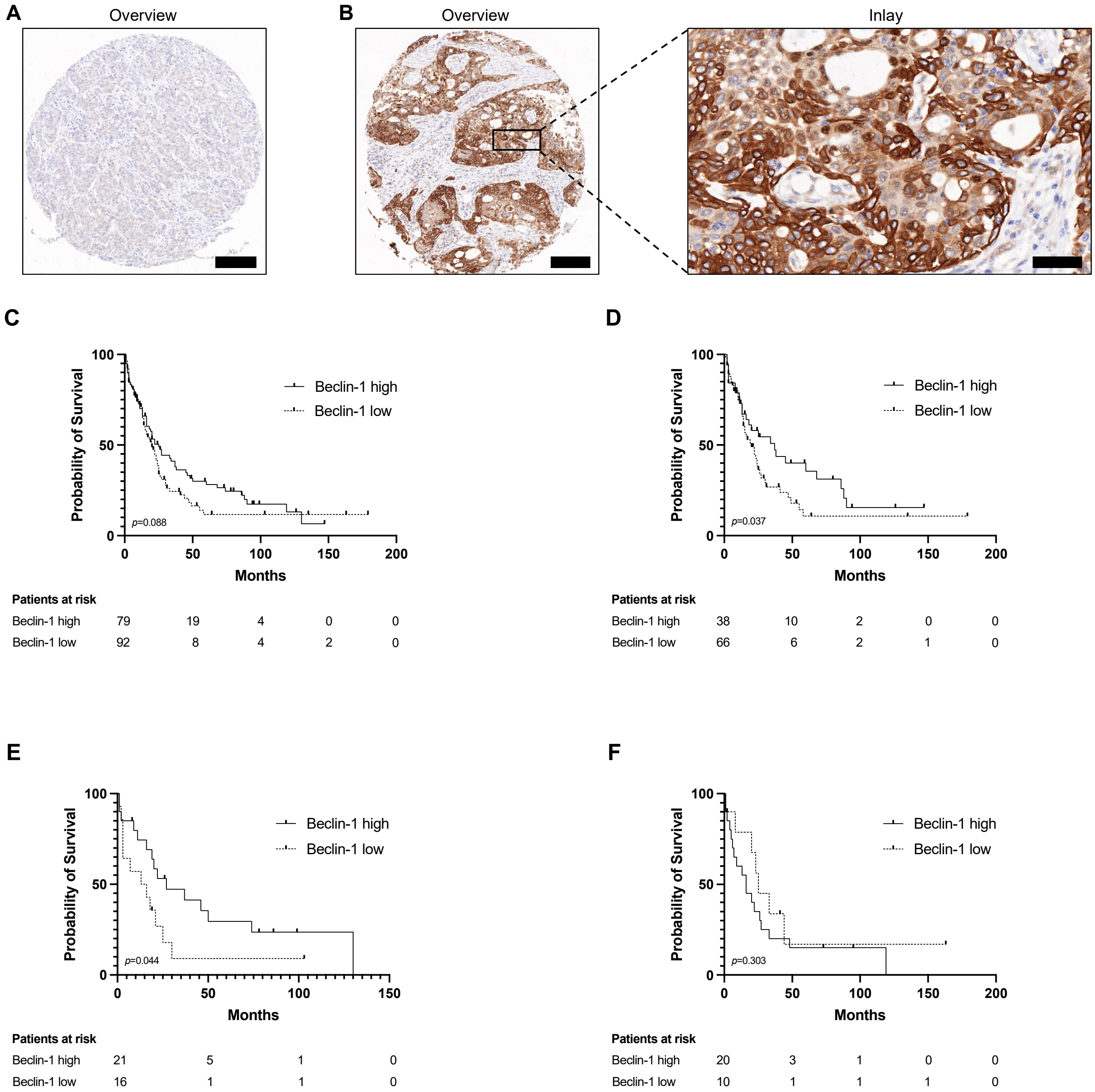

Figure S1. Representative images of Beclin-1 antibody staining in CCA tissue and corresponding Kaplan Meier curves for overall survival. (**A/B**) Representative immunohistochemistry of low (**A**) and high (**B**) expression of Beclin-1 in TMA cores of CCA patients. Original magnification x8.5 for overview and x40 for inlay, respectively. Scale bars: 200 µm for overview and 50 µm for inlay, respectively. (**C-F**) Overall survival investigated for Beclin-1 in all types of CCA (**C**), iCCA (**D**), pCCA (**E**) and dCCA (**F**). Date of last follow-up was treated as censored observation. 171 CCA patients were eligible for immunohistochemical assessment of Beclin-1. Abbreviations: Cholangiocarcinoma (CCA), intrahepatic Cholangiocarcinoma (iCCA), distal Cholangiocarcinoma (dCCA), perihilar Cholangiocarcinoma (pCCA), Tissue Microarray (TMA).

Supplementary Tables

Table S1. The most differentially expressed genes among iCCA groups of active vs. non-active state of autophagy.

Genes	Log2 fold change	*p*-Value	Gene Sets
APOC3	-4.7	0.0322	PPAR signaling
ARG1	-4.18	0.0287	Myeloid Immune Evasion
APOB	-3.71	0.0107	TLR Signaling
FGA	-3.49	0.000521	TLR Signaling
CYP8B1	-3.44	0.0489	PPAR signaling
ADH6	-3.33	0.00243	Fatty Acid Metabolism. Glycolysis and Glucose Import
TNFSF9	-3.13	0.0014	Anergy. T Cell Checkpoint Signaling. T Cell Exhaustion
ELOVL2	-3.04	0.000161	Fatty Acid Metabolism
AK4	-2.77	0.0017	Hypoxia Response
HDAC9	-2.69	0.00348	Epigenetic Modification. Notch Signaling
HGF	-2.49	0.0142	IL-7 Signaling. MAPK Signaling. Other Interleukin Signaling.
			PI3K-AKT Pathway
MIF	-2.32	0.00471	Other Interleukin Signaling
SNAI2	-2.19	0.0113	Epigenetic Modification
CCNB1	-2.14	0.0114	Cell Cycle. Epigenetic Modification. Senescence & Quiescence
LEPR	-2.11	0.00801	JAKSTAT Signaling
XAF1	-2.1	0.0363	Type I Interferon
IL18	-2.07	0.0151	IL-1 Signaling. IL-10 Signaling. NK Activity
UHRF1	-1.97	0.0172	Epigenetic Modification
TTK	-1.91	0.0186	Cell Cycle
CDC20	-1.85	0.0269	Cell Cycle
CHEK1	-1.84	0.00338	Cell Cycle. Epigenetic Modification. Senescence & Quiescence
PTGR1	-1.84	0.0129	NK Exhaustion
ECHS1	-1.74	0.0194	Fatty Acid Metabolism

Genes	Log2 fold change	*p*-Value	Gene Sets
BAK1	-1.73	0.0212	Apoptosis
ADORA2B	-1.73	0.0215	Myeloid Immune Evasion
RORgt	-1.7	0.0414	Other Interleukin Signaling
PSMA6	-1.69	0.0189	Antigen Presentation. BCR Signaling
JUN	-1.67	0.0311	AP-1 Signaling. Notch Signaling
IFIT1	-1.62	0.0428	Type I Interferon
DRAP1	-1.61	0.0255	TGF-beta Signaling
UBE2I	-1.6	0.0219	NF-kB Signaling
MCM7	-1.54	0.0243	Cell Cycle
EHHADH	-1.51	0.0267	Fatty Acid Metabolism. PPAR signaling
TICAM1	-1.48	0.0232	NF-kB Signaling. NK Activity. TLR Signaling
ALDOA	-1.48	0.0272	Glycolysis and Glucose Import. Hypoxia Response
CAMK2D	-1.47	0.0407	Epigenetic Modification. Hypoxia Response. Type II Interferon
CEBPB	-1.47	0.0421	AP-1 Signaling. Senescence & Quiescence. TNF Signaling
CHRD	-1.46	0.0346	TGF-beta Signaling
SMAD3	-1.43	0.027	Notch Signaling. Senescence & Quiescence. TGF-beta Signaling
TNFRSF1A	-1.42	0.0249	IL-10 Signaling. MAPK Signaling. mTOR Signaling. NF-kB Signaling. TNF Signaling
YWHAZ	-1.42	0.0302	Cell Cycle. Notch Signaling. Other Interleukin Signaling. PI3K-AKT Pathway
BAX	-1.39	0.008	Apoptosis
TELO2	-1.38	0.048	mTOR Signaling
RBX1	-1.37	0.046	Antigen Presentation. Cell Cycle. Hypoxia Response. IL-1 Signaling. Notch Signaling. TGF-beta Signaling
BID	-1.36	0.0183	Apoptosis. Cytotoxicity
DHRS3	-1.36	0.0445	RAR Signaling
ACSL3	-1.35	0.0292	Fatty Acid Metabolism. PPAR signaling

Genes	Log2 fold change	*p*-Value	Gene Sets
LDHA	-1.34	0.0177	Anergy. Glycolysis and Glucose Import. Hypoxia Response
RRAS	-1.33	0.0269	MAPK Signaling. Senescence & Quiescence
BMP2	-1.32	0.0232	TGF-beta Signaling
BCL2L1	-1.29	0.0333	Apoptosis. PI3K-AKT Pathway
MCL1	-1.29	0.0496	Apoptosis. JAKSTAT Signaling. Other Interleukin Signaling. PI3K-AKT Pathway
NRAS	-1.23	0.0411	MAPK Signaling
ORC2	-1.22	0.037	Cell Cycle
CASP8	-1.2	0.0362	Apoptosis. NK Activity. TLR Signaling. TNF Signaling
PSMC6	-1.14	0.037	Antigen Presentation. BCR Signaling
NPRL3	-1.14	0.0496	mTOR Signaling
ACOT1/2	-1.12	0.0315	Fatty Acid Metabolism
TNFSF10	-1.11	0.0438	Apoptosis. Cytotoxicity. NK Exhaustion
VDAC1	-1.07	0.048	Senescence & Quiescence
SPAG9	-1.06	0.0263	AP-1 Signaling
GOT2	-1.05	0.033	Glutamine Metabolism
ACAT2	-1.05	0.0456	Fatty Acid Metabolism
PSMA1	-1.01	0.0443	Antigen Presentation. BCR Signaling
TRIM33	-1.01	0.047	TGF-beta Signaling
PECAM1	1.03	0.0431	T Cell Checkpoint Signaling
TOX	1.67	0.0205	NK Activity
TPSAB1/B2	2.53	0.00544	Proteolysis
IL6	3.57	0.00091	IL-6 Signaling

'Estimated log fold-change' estimates a gene's differential expression. For categorical covariates, a gene is estimated to have 2^(log fold change) times its expression in non-active autophagy baseline samples, holding all other variables in the analysis constant. The log2 and linear fold change is also presented along with a *p*-Value.

Table S2. Top Genes from all Clusters associated to 9 representative terms and pathways using REACTOME_pathways ontology database in addition to Figure 5.

GOID	GOTerm	% Associated Genes	Nr. Genes	Associated Genes Found
R-HSA:69481	G2/M Checkpoints	4.79	8.00	CCNB1. CHEK1. MCM7. ORC2. PSMA1. PSMA6. PSMC6. YWHAZ
R-HSA:109581	Apoptosis	6.04	11.00	BAK1. BAX. BCL2L1. BID. CASP8. PSMA1. PSMA6. PSMC6. TICAM1. TNFSF10. YWHAZ
R-HSA:5357801	Programmed Cell Death	5.61	12.00	BAK1. BAX. BCL2L1. BID. CASP8. IL18. PSMA1. PSMA6. PSMC6. TICAM1. TNFSF10. YWHAZ
R-HSA:1280215	Cytokine Signaling in Immune system	2.75	20.00	BCL2L1. CAMK2D. CASP8. HGF. IFIT1. IL18. IL6. JUN. MCL1. MIF. NRAS. PSMA1. PSMA6. PSMC6. RBX1. SMAD3. TNFRSF1A. TNFSF9. XAF1. YWHAZ
R-HSA:168256	Immune System	1.33	27.00	ALDOA. APOB. ARG1. BCL2L1. CAMK2D. CASP8. CDC20. FGA. HGF. IFIT1. IL18. IL6. JUN. MCL1. MIF. NRAS. PECAM1. PSMA1. PSMA6. PSMC6. RBX1. SMAD3. TICAM1. TNFRSF1A. TNFSF9. XAF1. YWHAZ
R-HSA:449147	Signaling by Interleukins	3.16	15.00	BCL2L1. CASP8. HGF. IL18. IL6. JUN. MCL1. MIF. PSMA1. PSMA6. PSMC6. RBX1. SMAD3. TNFRSF1A. YWHAZ
R-HSA:5663202	Diseases of signal transduction by growth factor receptors and second messengers	2.77	12.00	BCL2L1. CAMK2D. CEBPB. FGA. HDAC9. HGF. NRAS. PSMA1. PSMA6. PSMC6. RBX1. SMAD3

GOID	GOTerm	% Associated Genes	Nr. Genes	Associated Genes Found
R-HSA:5683057	MAPK family signaling cascades	3.38	11.00	BCL2L1. CAMK2D. FGA. HGF. IL6. JUN. NRAS. PSMA1. PSMA6. PSMC6. RBX1
R-HSA:5684996	MAPK1/MAPK3 signaling	3.50	10.00	[BCL2L1. CAMK2D. FGA. HGF. IL6. NRAS. PSMA1. PSMA6. PSMC6. RBX1]

ClueGO LOG: Genes from Cluster#1: unique uploaded ids 69 with 3 (4.35%) missing. 66 Genes with functional annotations in all selected ontologies from Cluster#1: 62 (93.94%). 4 (6.06%) are not functionally annotated in any selected ontology. Genes from all clusters associated to 30 representative terms and pathways (after applying general selection criteria): 62 (93.94%). Genes from all clusters associated to 9 representative terms and pathways (after *p*-Value significance selection criteria): 37 (56.06%). REACTOME_Pathways_15.11.2022. Statistical test used = enrichment/depletion (two-sided hypergeometric test). Use *p*-Value cutoff = true. *p*-Value cutoff = 1.0E-4. Correction method used = Bonferroni step down. Min GO level = 2. Max GO level = 5.
